# Matching DMFT calculations with photoemission spectra of heavy fermion insulators: universal properties of the near-gap spectra of SmB_6_

**DOI:** 10.1038/s41598-017-12080-5

**Published:** 2017-09-20

**Authors:** Chul-Hee Min, F. Goth, P. Lutz, H. Bentmann, B. Y. Kang, B. K. Cho, J. Werner, K.-S. Chen, F. Assaad, F. Reinert

**Affiliations:** 1 Experimentelle Physik VII and Röntgen Research Center for Complex Materials (RCCM), Universität Würzburg, 97074 Würzburg, Germany; 2 Institut für Theoretische Physik und Astrophysik, Universität Würzburg, 97074 Würzburg, Germany; 3 School of Materials Science and Engineering, Gwangju Institute of Science and Technology (GIST), Gwangju, 61005 Korea

## Abstract

Paramagnetic heavy fermion insulators consist of fully occupied quasiparticle bands inherent to Fermi liquid theory. The gap emergence below a characteristic temperature is the ultimate sign of coherence for a many-body system, which in addition can induce a non-trivial band topology. Here, we demonstrate a simple and efficient method to compare a model study and an experimental result for heavy fermion insulators. The temperature dependence of the gap formation in both local moment and mixed valence regimes is captured within the dynamical mean field (DMFT) approximation to the periodic Anderson model (PAM). Using the topological coherence temperature as the scaling factor and choosing the input parameter set within the mixed valence regime, we can unambiguously link the theoretical energy scales to the experimental ones. As a particularly important result, we find improved consistency between the scaled DMFT density of states and the photoemission near-gap spectra of samarium hexaboride (SmB_6_).

## Introduction

The interplay of topology and correlation effects has led to generalizations of the concept of topological insulators^1,2^, namely to symmetry protected topological surface states (TSS)^3^. Topological Kondo insulators (TKI)^4,5^ are an example of a time reversal symmetry protected TSS. Here, correlation effects are dominant in the formation of the low-energy quasiparticle excitations, but the ground state itself is believed to be adiabatically connected to a Fermi liquid. In this sense, one can construct a link to a non-interacting fermion system (with heavy mass), and take over our understanding of the ${{\mathbb{Z}}}_{2}$ classification^6^ to this class of correlated materials^7–11^. A well-known candidate for the TKI is the compound samarium hexaboride (SmB_6_). It is a paramagnetic bulk insulator^12,13^ with a smooth gap opening consisting of 4*f* and 5*d* characters^14–16^.

The microscopic picture of this particular coherent state has been qualitatively captured with various mean-field approximations to the periodic Anderson model (PAM)^4,9,14,17–34^. However, a quantitative comparison between theoretical and experimental density of states (DOS) is hampered by the complexity of the actual material and by the experimental processes, *e*.*g*. surface reconstructions, multiplet structure, line broadening, information depth, etc^35–37^. Moreover, such quantitative analyses require to identify the validity range of the theoretical results applicable to the experimental data. Hence, it is in general necessary to find a proper energy conversion between theoretical and experimental energies.

The aim of this paper is to present a new perspective on the comparison of theoretical and experimental DOS of heavy fermion (HF) insulators. In particular, we focus on the dynamical emergence of the hybridization gap of topological HF insulators in units of the coherence temperature *T*_coh_. This scaling provides an effective way to convert the theoretical to the experimental energy scales. Such scaling approaches have been mainly applied to transport properties of metallic Ce- and Yb-based HF systems^38,39^. For these metallic cases, there is a certain freedom in defining the quantitative *T*_coh_ depending on the respective physical properties^40–42^. This is due to the nature of the crossover in PAM (See also the Sec. I of the Supplementary Information). However, this definition is less ambiguous for the insulating phase. The reason is that most physical properties will drastically change with the gap opening. Thus, *T*_coh_ can be chosen to mark the temperature at which the gap opens, and can therewith be connected to the topological phase by monitoring the emergence of the TSS^24,25,28^.

Since the emergent topological band structure is nothing else but evidence for coherence, we can use the net polarization of the TSS to define *T*_coh_ in the model calculations^43^. One can define a pseudo-spin Hall conductivity that takes the value of unity at zero temperature in the insulating state, thereby defining a topological index *N*_2_^24,25^. The topological coherence temperature *T*_N_ corresponds to the *T* scale at which the index *N*_2_ takes the value of one-half, *i*.*e*. the pseudo-spin Hall conductivity of the TSS takes half of its zero temperature quantized value. With the definition of *T*_N_, a unifying crossover parameter can be defined to describe both the lattice coherence and the topological phase. We revisit the physical consequences occurring at the *T*_N_ values, which are carefully evaluated in the refs^24,25^. considering the index *N*_2_ with the same input parameters used in this study. In particular, we analyzed the gap evolution and occupation numbers to extract the experimental results.

Our choice for the theoretical approach is the dynamical mean field theory (DMFT)^24^. Since we take a simple version of the PAM, our results cannot effectively capture every spectral detail of SmB_6_^33^. Nevertheless, a simplified model has the advantages of surveying unique and general properties in the wide ranges of input parameters. In previous studies, the electron correlation *U* was tuned from the mixed valent regime to the local moment regime, and we identified universal features in the *T*-dependent *N*_2_ index^21,24^. Experimentally, we investigated the near-gap region of SmB_6_ by photoemission spectroscopy (PES). There are fascinating low-*T* properties observed in SmB_6_^44–57^, which we do not consider in the present study. Note that, as the energy resolution of the PES setup is limited, PES results have not detected all degenerate *f* bands predicted in first-principle calculations^7,8,17,27^ in the energy range of 20 meV below the chemical potential^22,58–63^. Instead, we focus on the gap opening of SmB_6_ that is observed in the PES experiments and explains various transport properties near 50 K^22,62^.

We will first show our theoretical results near *T*_N_ and place emphasis on the temperature dependence of the orbital dependent occupation numbers. In the following section, we display the scaled DMFT spectra at different *U* values, and clarify the universal gap features. Adopting the *T*_N_ scaling, we will show consistent results on the gap evolution obtained from theory and experiment. In the discussion section, the importance of the scaling with *T*_N_ is described and the key parameters are discussed. In the supplementary information, we give a detailed discussion of the crossover temperature in the Anderson model, additional noteworthy points for the comparisons, and illustrate the detailed spectral weight redistribution in the *E*(*k*) plot near *T*_N_. Although our simplified model captures the overall T-dependence of the electronic structure, it fails to properly describe material specific aspects, e.g. in present case, the slight slope change in Sm valence at 120 K or the slight increase below 20 K^64^ are not realized in our result.

## Results

### *T*-dependence of theoretical DOS and occupation numbers

**Figure 1:**
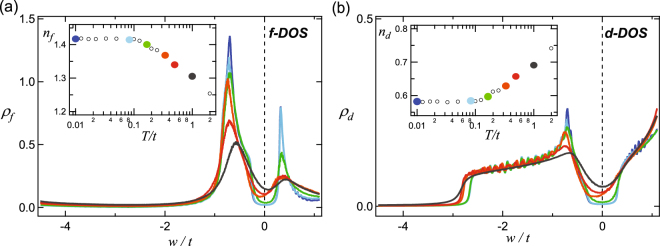
As an example of the DMFT results, temperature (*T*) dependence for density of *f* and *d* states, for the mixed valence regime (*U*/*t* = 5) is shown in (**a**) and (**b**), respectively. The coherence temperature for this regime is  *T*_N_/t = 0.2125, which is evaluated from index *N*_2_. The energy is normalized to the hopping parameter *t*. In the same colors used for density of states (DOS) lines, the corresponding occupation numbers for *f* and *d* states are shown as a function of *T/t* in the insets. The gap, whose character mainly originates from *f* states at high *T*, becomes clearer and deeper with decreasing *T*. Note that at *T* ≤ *T*_N_ the spectral weights inside the gap, and the occupation numbers of *f* and *d* states start to saturate (insets).

Figure 1 shows the *T*-dependence of the *f* and *d* density of states (DOS) as a function of energy over hopping parameter for the *d* states (*w*/*t*)^24^. Here, we illustrate the DOS near *T*_N_/*t* = 0.2125 in the mixed valence regime (*U*/*t* = 5). In the insets, the occupation numbers *n*_f_ and *n*_d_ of each DOS are depicted as a function of *T*/*t* in the colors corresponding DOS lines. Figure 1(a) shows the occupied *f* -DOS near *E*_F_, which is related to the Hubbard band lying near the chemical potential, and Fig. 1(b) mainly shows the occupied *d* -DOS, which originates from the 2D conduction band. Both *f*- and *d* - DOS are only broadened due to the imaginary part of the self-energy. (The wiggling features in Fig. 1(b) are reminiscent of the finite *k*-mesh used in the DMFT calculations). The spectral weight in the gap region (|*w*/*T*_*N*_| ≤ 1) is mainly of *f* character, and clearly reduces with decreasing temperature, being qualitatively consistent with the PES spectra so far^59,62,65–68^.

The results in units of *t* (Fig. 1) do not match with the experimental spectra. For example, if *t* ∼ 1 eV, *U* becomes 5 eV, *T*_*N*_ becomes 0.2125 eV ∼ 2500 K, and the maximum width of the *f* peak becomes 1 eV. Moreover, the total energy resolution (ΔE) of the experimental setup should be considered in the DMFT spectra to fairly compare each other, which is not feasible with this energy scale. Hence, it is difficult to compare with experimental spectra quantitatively. Interestingly, with the assumption of *t* ∼ 1 eV, both *T*_coh_ and the peak width are about two orders of magnitude higher than the PES results of SmB_6_^62,65^. Hence, a proper scaling factor might exist and provide the connection between theoretical and experimental energy scales. In order to extract *T*_coh_ in the experiment data (${T}_{{\rm{coh}}}^{exp}$) corresponding to the *T*_N_, we first look carefully at the unique *T* dependence of *n*_f_ (Fig. 1 (a, inset)). Below *T*_N_, *n*_f_ and *n*_d_ are almost saturated to the maximum and minimum occupations, respectively. Secondly, we recognize that the intensity at *E*_F_ is saturated in both *f*- and *d*- DOS (green lines) below *T*_N_.

Based on the two signatures around *T*_N_, we search for ${T}_{{\rm{coh}}}^{exp}$ in SmB_6_ as determined from experiments. According to the saturation point in the *T*-dependent Sm valence obtained from the XAS result^64^, the ${T}_{{\rm{coh}}}^{exp}$ should be around 50 K. Recent 4*f* character-sensitive PES studies show that the gap opening happens below 60 K^22,62^. Furthermore, various other experiments revealed a similar characteristic temperature of ∼50 K^12,13,45,47,51,64,69–82^. Thus, it is reasonable to define a characteristic temperature out of the PES spectra related to the gap formation of the HF insulator, and connect the theoretical energy scale to the experimental energy and temperature as follows: *w*/*T*_N_ = *E*/(*k*_*B*_ ${T}_{{\rm{coh}}}^{exp}$), and *T*/*T*_N_ = ${T}^{\exp }/{T}_{{\rm{coh}}}^{exp}$ where *k*_*B*_ is the Boltzmann constant. The peak broadening due to the experimental resolution can be also implemented in the theoretical spectra. Thus, the theoretical DOS are broadened in energy *w* by *T*_N_ ⋅ (7 meV)/(*k*_*B*_ ⋅ 50 K).

### Universal gap evolution for *U*/*t* 5, 6, 7, and 8

**Figure 2:**
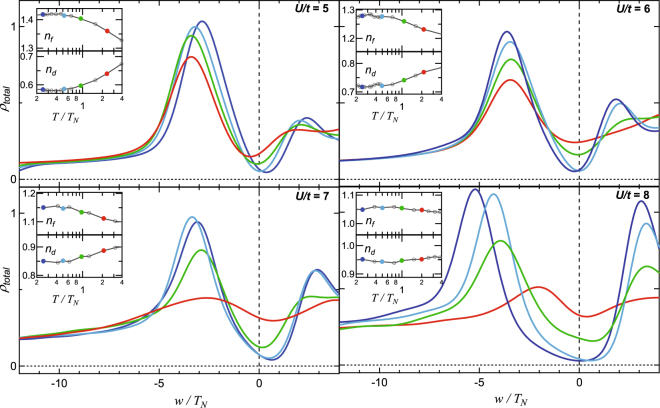
Universal gap evolution studied for *U*/*t* = 5, 6, 7; and 8. Total DOS, which are exactly treated as the spectra in Fig. 3(b). The spectral weight in the gap region decreases with decreasing *T*. The gap region becomes clearer. Insets show the *T* dependence of the occupation numbers of *n*_f_ and *n*_d_ for each *U* values. The occupation numbers vary as a function of *T* strongly in the mixed valence regime (*U*/*t* = 5). But, the variation reduces continuously from the mixed valence regime to the local moment regime (*U*/*t* = 8), which indicates a crossover.

In order to demonstrate the universal property of the model applying the *T*_N_-scaling, total DOS, which is the sum of *f*- and *d*- DOS, near the gap region are shown for various *U*/*t* = 5, 6, 7 and 8, in Fig. 2. Estimated *T*_N_/*t*, values for *U*/*t* = 5, 6, 7 and 8 are 0.2125, 0.155, 0.0795, and 0.0263, respectively^25^. With increasing *U*/*t* from 5 to 8, T_N_ decreases by an order of magnitude. Note that after the scaling with the respective *T*_N_, the comparison of the gap openings becomes possible for various *U/t*. In all spectra, the *f* peak appears in the range of −5 ≤ *w*/*T*_N_ ≤ −3, and the spectral weight of the gap region decreases with lowering temperature. The gap opening in both mixed valence and local moment regimes is similar to each other. This is the unique characteristics of the HF insulator, showing the gap evolution is universal, *i*.*e*. mostly independent from the actual set of model parameters. The insets of Fig. 2 illustrate the *T* dependence of *n*_f_ and *n*_d_ for various *U/t* values. Normalizing *T* by *T*_N_ leads to the universal change in the occupation numbers. Below *T*_N_, the occupation numbers saturate to a constant value in all cases. The difference in the high and low *T*﻿ values of *n*_*f*_ and *n*_d_ decreases when *U*/*t* increases. It is recognizable till *U/t* = 7, where the n_f_ is slightly larger than 1.1.

Four main characteristics are found in the DMFT spectra as a function of *U*/*t*, which become obvious after the scaling and taking care of the resolution. First, a systematic energy shift in the gap minimum apparently appears in the mixed valence regime, *i*.*e*. *U*/*t* = 5. Second, at higher *U*/*t*, the gap deepens more rapidly with respect to the scaled temperature. Third, at the converted temperature of *T* ∼ 2*T*_*N*_ (red lines), the gap minimum positions at slightly different energies for different *U/t*. The gap minimum appears below *E*_*F*_ at *U*/*t* = 5, but it appears at higher energy with increasing *U/t*. Fourth, the *f* peak intensity at high temperatures is obviously higher in the mixed valence regime because of the presence of the atomic multiplet ^6^ *H*_5/2_ state. Thus, although the lattice coherence has not been developed, it is still possible to observe the peak structure near *E*_*F*_^35,83^.

### Mixed valence: PES vs. DMFT for *U*/*t* = 5

**Figure 3:**
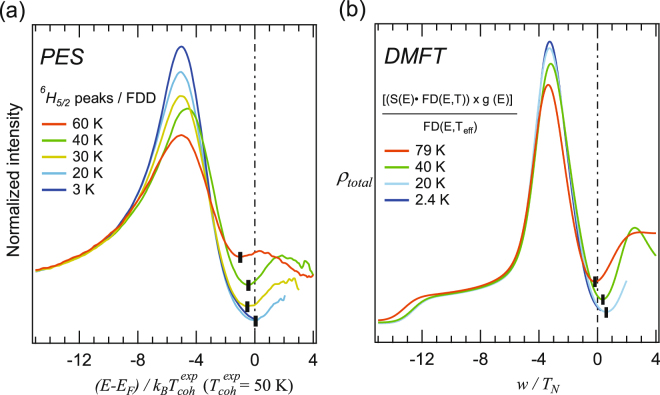
Comparison of the PES spectra of SmB_6_ with the theoretical DOS after scaling with the corresponding *T*_coh_. (**a**) The angle-integrated spectra of SmB_6_ are divided by the Fermi-Dirac function^62^. The energy axis is reduced by the coherence scale determined from experiments (${T}_{{\rm{coh}}}^{exp}$ = 50 *K*). (**b**) Reconstructed spectra from theoretical calculation, considering Fermi-Dirac distributions and total experimental resolutions (see text), in order to compare with the experiment spectra (**a**). In unit of the respective coherence energies, the *f* peaks in (**a**) and (**b**) appear at the energies of the same order of magnitude. Moreover, the gap regions show similar *T* dependence such that the gap minimum (black rectangles) shifts toward high energy with decreasing *T*.

Figure 3(a) shows the angle-integrated photoemission spectra of SmB_6_ divided by the Fermi-Dirac distribution (FDD)^62^ on the reduced energy scale of ${T}_{{\rm{coh}}}^{\exp }$. The peak at (*E*-*E*_*F*_)/(*k*_*B*_ ${T}_{{\rm{coh}}}^{exp}$) = −5 is the 4*f* multiplet excitation (^6^ *H*_5/2_) with lowest binding energy. The gap in the PES data is getting deeper with decreasing *T*, comparable to the behavior observed in the theoretical DOS (as shown in the previous figures). We compare now the experimental and the theoretical spectra in more detail using their functional relation to the scaling. If SmB_6_ has the scaling property, the energy and *T* in the PES spectra can be rescaled to link with the DMFT energy.

To make the calculated DOS directly comparable to the experimental spectrum, we treated the DOS by an established numerical procedure already successfully applied to metallic heavy fermion systems^84^. The total DOS was multiplied by the FDD, and convoluted by a Gaussian function regarding the total resolution. Lastly, the resulting spectrum was normalized to the FDD as shown in Fig. 3(b) in order to get comparable spectra in Fig. 3(a).

As a result, the scaled theoretical and experiment spectra show a surprisingly good agreement in the *T* dependence of both the 4*f* peak and the gap opening. As already theoretically suggested^24,25^, the *4f* energy positions, the line widths of the peaks, and the size of the gap are on the scale of the respective *T*_coh_. In particular, both gap developments near *T*_coh_ are very similar. The gap minima in both theory and experiment, marked with black rectangles in Fig. 3, shift from below *E*_F_ toward high energy with elaborating *T*. Note that when the accuracy of our DMFT results is tested, we find that the gap minima are among the most reliable features in the spectra.

## Discussion

Heavy fermions are a canonical example of a multi-scale problem. The bare scales such as the Coulomb repulsion *U* and conduction electron band width are of several eV. In contrast, the emergent scales, in particular the coherence scale, are measured in units of meV^85^. A realistic multi-scale calculation is prohibitively difficult since it would have to take into account the details of the orbital structure and interactions. Low energy physics can however show signs of universality in the sense that scaling with the appropriate energy scale allows comparison between different experimental realizations of the phenomena under consideration and with theory. Our study follows precisely this idea. A good account of the low energy behavior of the photoemission spectra can be obtained by scaling the experimental and theoretical data with the corresponding coherence scale and making sure that the theoretical calculations are well in the mixed valence regime^21,24^.

Our theoretical model is a PAM on a square lattice^21,24^, which we solve by means of DMFT that maps the lattice problem onto a single Anderson impurity model (SIAM). The DMFT approximation captures the salient many body physics of the paramagnetic phase of heavy fermions^86^. Due to the locality of the approximation only dynamical fluctuations are relevant for the emergence of the coherence scale^41^. In this sense we do not expect the spacial degrees of freedom to play a dominant role in the analysis of the temperature dependence of the low energy DOS. In particular in the SIAM, dimensionality enters only in terms of the bare density of states at the Fermi energy and is one of the parameters that determine the Kondo scale.

In our model study we consider a two fold degenerate *f* band unlike in refs^7,8,20^. The detailed band structure and its symmetry aspects are very interesting issues in the rare-earth hexaborides^17^, which will help to find alternative topological Kondo insulators. Nevertheless, in our model we treat only the lowest-lying two bands, *i*.*e*. one *f* band and one *d* band, in order to capture the general gap evolution. As shown in Fig. 3, the line broadening due to the limited experimental resolution makes the theoretical *f* peak width comparable to the experimental one (See also the Sec. II of the Supplementary Information for further discussion). Thus, it again appears that our simple model is able to capture the main low-energy features of the DOS.

Hence, this demonstrates that just the universal scales of model calculations are sufficient to reproduce the general gap opening appearing in the experiment. In order to obtain further agreement, only few parameters should be adjusted to realize, in particular, the *T* dependence of the gap evolution and the occupation numbers. These two features are the most reliable and sensitive characteristics in our theoretical results, which mainly depend on the configuration of the input parameter set, *i*.*e*. the distinction between local moment and mixed valence regimes. Among our survey parameters, the mixed valence configuration gives the best agreement. Hence, our investigation demonstrates that the key parameters, namely the *coherence temperature* and the *degree of valence mixing*, suffice to realize the experiments. Our finding actually proves that the gap nature of SmB_6_ involves the emergence of the coherent renormalized bands in the framework of the Fermi liquid theory. Therefore, the topological phase of SmB_6_ can be classified with the topological indices applicable to non-interacting insulators as presumed in various theoretical studies^4,10,11^.

## Methods

Theoretical studies on the *T* dependence of the electronic structure of heavy fermion insulators (half-filled cases) in 2D square lattice were carried out based on the DMFT^87^, which maps a 2D model onto an auxiliary impurity problem. The impurity problem is then solved by the the numerically exact CT-HYB quantum Monte Carlo algorithm. For the simple model study, we use tight binding bands with the same input parameters as in the ref.^24^, which are normalized with the conduction hopping parameter *t*, *e*.*g*. 5.0 ≤ *U*/*t* ≤ 8.0, *ε*_*f*_/*t* =  −6.0, the hybridization *V*/*t* = 0.4, etc. The high resolution photoelectron spectroscopy (PES) experiments were carried out at the UE112-PGM-1b (“1^3^”) beamline of BESSY II using a Scienta R4000 analyzer at 3 K ≤  *T* ^*exp*^ ≤ 60 K^62^. The excitation photon energy was *hν* = 70 eV, whose constant energy map at *E*_F_ covers *k*_*z*_ = 6*π*/*a* in normal emission, with the high energy resolution of 7 meV. Detailed experimental conditions can be found in ref.^62^.

## Electronic supplementary material

Supplementary information for matching theoretical and experimental DOS
