# Supplementary material for: Matching DMFT calculations with photoemission spectra of heavy fermion insulators: universal properties of the near-gap spectra of SmB6
Source: Sci Rep. 2017 Sep 20;7:11980. doi: 10.1038/s41598-017-12080-5 (PMC5607333; doi:10.1038/s41598-017-12080-5)
Supplement: Supplementary file 1 — Supplementary information for matching theoretical and experimental DOS [file 41598_2017_12080_MOESM1_ESM.pdf]

Supplementary Information for Matching DMFT calculations with photoemission spectra of heavy fermion insulators: universal properties of the near-gap spectra of SmB_6

Chul-Hee Min,* P. Lutz, H. Bentmann, and F. Reinert

Experimentelle Physik VII and Röntgen Research Center for Complex Materials (RCCM), Universität Würzburg, 97074 Würzburg, Germany

F. Goth, J. Werner, K.-S. Chen, and F. Assaad

Institut für Theoretische Physik und Astrophysik, Universität Würzburg, 97074 Würzburg, Germany

B. Y. Kang and B. K. Cho

School of Materials Science and Engineering, Gwangju Institute of Science and Technology (GIST), Gwangju 61005, Korea.

(Dated: February 23, 2017)

* corresponding author. Email:cmin@physik.uni-wuerzburg.de

I. FERMI LIQUID PHASE IN THE ANDERSON MODEL AND CHARACTERISTIC TEMPERATURES

The basic understanding of the renormalized f states is substantially established in the local moment regime of the single impurity Anderson model (SIAM). When the local moments of localized f states are antiferromagnetically coupled with conduction electron states, they become quasiparticle states in the Landau Fermi liquid scheme. This local Fermi liquid phase emerges as a crossover, which smoothly happens without any phase transition, *i.e.* a crossover [1–3]. Moreover, the physical properties follow universal functions on one energy scale, which is the Kondo temperature T_K. It implies that the physical properties can be written in terms of T/T_K [4]. Other crossover parameters for the paramagnetic ground states in the periodic Anderson model (PAM) have also been studied when the screened local moments, which are positioned on a lattice, form coherent Bloch-like states [1, 5–10]. The coherent states can be clearly identified below the coherence temperature T_{coh}, which is typically smaller than T_K [7, 8]. Because the PAM may result in different ground states and phases, there are different characteristic temperatures [3]. However, for a simple Fermi liquid quasiparticle state, the concept of T_{coh} describes the emergence of the coherent quasiparticle band, especially for the *mixed valence* regime [1, 2, 10]. In this regime, the local moments are not clearly defined and charge fluctuations play an important role alongside spin fluctuations. Due to the charge fluctuations, spectral weight is redistributed across the gap, which has been experimentally and theoretically observed for SmB_6 [10–12].

II. EXPERIMENTAL PREREQUISITES

Note that the comparison of DMFT and PES results relies on the following three points: First, we have restricted the photon energy $h\nu > 40\,\mathrm{eV}$ to emphasize the $4f$ character in the photoemission spectra, which takes into account the dominant spectral weight of f states near gap region in the model study. Second, we have considered only the state $^6H_{5/2}$ appearing at the lowest energy below E_{F}. Experimentally, the next lowest state $^6H_{7/2}$, which appears at the energy of E-$E_{\mathrm{F}} \approx -0.18\,\mathrm{eV}$ [13–16], shows much less changes in both shape and intensity than $^6H_{5/2}$, and thus shall be neglected for the contribution to low-temperature behavior. And, third, to make connection with the DMFT calculation, we have chosen model

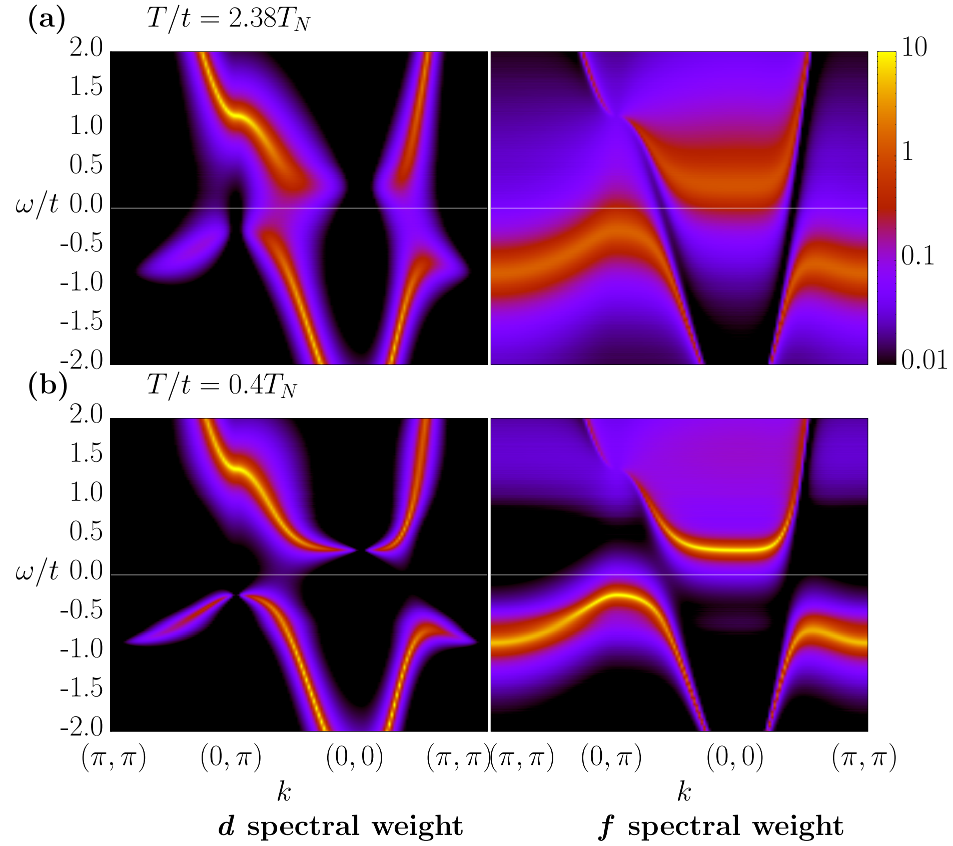

Figure S1. (Color online). Single-particle spectral functions in the mixed valence regime above and below the topological coherence temperature T_{N}. Left and right panels show the conduction d and localized f spectral weight, respectively. (a) Above T_{N}, the f spectral weights are strongly diffused. The blurred f features strongly contribute to the total spectral weight in the gap region. (b) Below T_{N}, the f spectral weight in the gap region is dramatically reduced, and the f dispersion becomes clearer. Moreover, the dispersions of the d and f features become identical, which is a result of the strong character mixing.

parameters to reproduce T-dependence in the occupation numbers in SmB_6, *i.e.* the occupancies still vary at temperature near gap opening.

III. UNIQUE BAND DISPERSION OF THE MIXED VALENT INSULATOR

Fig. S1 shows how signatures of coherence appear in the mixed valent insulator by comparing the theoretical spectral functions at temperatures above and below T_{N}. The temperature dependent d and f spectral weights obtained from DMFT studies are shown on the left and

right panels, respectively (See Ref. [10, 17] for details). Two main changes appear as one crosses the temperature scale $T_{\rm N}$, Fig. S1 (a) and (b). One is that the gap is getting more pronounced, and the other is that the f and d spectra becomes more similar to each other. Note that the f features near $w/t \sim 0$ is apparent even at high T, so it is not buried under the background intensity (incoherent spectral weight). Thus, at high T the f bands in the mixed regime is not the same as the Kondo resonance in the local moment regime, which completely disappears at high temperatures.

Close to $E_{\rm F}$, the f features are getting blurred at $T > T_{\rm N}$ (Fig. S1 (a, right)), whereas they have reduced intensity at $T < T_{\rm N}$ (Fig. S1 (b, right)). The reduced spectral weight in the gap region has been already observed in other PES investigations [18–21]. In addition, the band dispersions of heavy f states and itinerant d states change as a function of the temperature. Above $T_{\rm N}$, d and f spectral weights are strongly dispersing and flat bands, respectively, whereas below $T_{\rm N}$ they show very alike band dispersion. This is due to hybridization, which gives rise to the character mixing of f and d states. Hence, if one can selectively measure one of the orbital character by setting adequate experimental conditions, it will be possible to observe the change in the band dispersion with temperature. Moreover, we note that in the model calculations the hybridization between the f and d states is given by $\boldsymbol{V}(\boldsymbol{k}) \cdot \boldsymbol{\sigma}$, where $\boldsymbol{\sigma}$ is a vector of Pauli spin matrices. Time reversal symmetry requires $\boldsymbol{V}(\boldsymbol{k})$ to be odd, such that the hybridization vanishes at time reversal momenta. This explains in particular the loss of spectral weight at the high symmetry points $(0,0)$, $(0,\pi)$ and (π,π) at low temperatures.

---

[1] F. D. M. Haldane, Phys. Rev. Lett. **40**, 416 (1978).

[2] J. Werner and F. F. Assaad, Phys. Rev. B **88**, 035113 (2013).

[3] C.-J. Kang, H. C. Choi, K. Kim, and B. I. Min, Phys. Rev. Lett. **114**, 166404 (2015).

[4] A. C. Hewson, *The Kondo Problem to Heavy Fermions* (Cambridge University Press, 1993).

[5] A. N. Tahvildar-Zadeh, M. Jarrell, and J. K. Freericks, Phys. Rev. Lett. **80**, 5168 (1998).

[6] R. Eder, O. Rogojanu, and G. Sawatzky, Phys. Rev. B **58**, 7599 (1998).

[7] S. Burdin, A. Georges, and D. R. Grempel, Phys. Rev. Lett. **85**, 1048 (2000).

[8] F. F. Assaad, Phys. Rev. B **70**, 020402 (2004).

[9] Y.-f. Yang, Z. Fisk, H.-O. Lee, J. D. Thompson, and D. Pines, Nature **454**, 611 (2008).

[10] J. Werner and F. F. Assaad, Phys. Rev. B **89**, 245119 (2014).

[11] J. D. Denlinger, J. W. Allen, J.-S. Kang, K. Sun, J.-W. Kim, J. H. Shim, B. I. Min, D.-J. Kim, and Z. Fisk, arXiv:1312.6637 [cond-mat] (2013).

[12] C.-H. Min, P. Lutz, S. Fiedler, B. Y. Kang, B. K. Cho, H.-D. Kim, H. Bentmann, and F. Reinert, Phys. Rev. Lett. **112**, 226402 (2014).

[13] J. N. Chazalviel, M. Campagna, G. K. Wertheim, and P. H. Schmidt, Phys. Rev. B **14**, 4586–4592 (1976).

[14] J. Denlinger, G.-H. Gweon, J. Allen, C. Olson, Y. Dalichaouch, B.-W. Lee, M. Maple, Z. Fisk, P. Canfield, and P. Armstrong, Physica B: Condensed Matter **281–282**, 716–722 (2000).

[15] J. D. Denlinger, J. W. Allen, J.-S. Kang, K. Sun, B.-I. Min, D.-J. Kim, and Z. Fisk, "Smb¡sub¿6¡/sub¿ photoemission: Past and present," in *Proceedings of the International Conference on Strongly Correlated Electron Systems (SCES2013)* (2014) Chap. 3, p. 017038, http://journals.jps.jp/doi/pdf/10.7566/JPSCP.3.017038.

[16] C.-H. Min, O. Sommer, B. Kang, B. Cho, H. Bentmann, and F. Reinert, Journal of Electron Spectroscopy and Related Phenomena **199**, 46 (2015).

[17] K.-S. Chen, J. Werner, and F. Assaad, Phys. Rev. B **90**, 115109 (2014).

[18] S. Souma, H. Kumigashira, T. Ito, T. Takahashi, and S. Kunii, Physica B: Condensed Matter **312–313**, 329–330 (2002).

[19] S. Nozawa, T. Tsukamoto, K. Kanai, T. Haruna, S. Shin, and S. Kunii, Journal of Physics and Chemistry of Solids **63**, 1223–1226 (2002).

[20] J. Jiang, S. Li, T. Zhang, Z. Sun, F. Chen, Z. R. Ye, M. Xu, Q. Q. Ge, S. Y. Tan, X. H. Niu, M. Xia, B. P. Xie, Y. F. Li, X. H. Chen, H. H. Wen, and D. L. Feng, Nat Commun **4** (2013), 10.1038/ncomms4010.

[21] N. Xu, C. E. Matt, E. Pomjakushina, X. Shi, R. S. Dhaka, N. C. Plumb, M. Radović, P. K. Biswas, D. Evtushinsky, V. Zabolotnyy, J. H. Dil, K. Conder, J. Mesot, H. Ding, and M. Shi, Phys. Rev. B **90**, 085148 (2014).
